# "What Do They Want Me To Say?" The hidden curriculum at work in the medical school selection process: a qualitative study

**DOI:** 10.1186/1472-6920-12-17

**Published:** 2012-03-26

**Authors:** Jonathan White, Keith Brownell, Jean-Francois Lemay, Jocelyn M Lockyer

**Affiliations:** 1 Department of Surgery, University of Alberta, Edmonton, Canada; 2 Departments of Medicine and Clinical Neurosciences, University of Calgary, Calgary, Canada; 3 Department of Pediatrics, University of Calgary, Calgary, Canada; 4 Department of Community Health Sciences, University of Calgary, Calgary, Canada

**Keywords:** Admissions, Medical school selection, Hidden curriculum, Conforming, Qualitative methods

## Abstract

**Background:**

There has been little study of the role of the essay question in selection for medical school. The purpose of this study was to obtain a better understanding of how applicants approached the essay questions used in selection at our medical school in 2007.

**Methods:**

The authors conducted a qualitative analysis of 210 essays written as part of the medical school admissions process, and developed a conceptual framework to describe the relationships, ideas and concepts observed in the data.

**Results:**

Findings of this analysis were confirmed in interviews with applicants and assessors. Analysis revealed a tension between "genuine" and "expected" responses that we believe applicants experience when choosing how to answer questions in the admissions process. A theory named "What do they want me to say?" was developed to describe the ways in which applicants modulate their responses to conform to their expectations of the selection process; the elements of this theory were confirmed in interviews with applicants and assessors.

**Conclusions:**

This work suggests the existence of a "hidden curriculum of admissions" and demonstrates that the process of selection has a strong influence on applicant response. This paper suggests ways that selection might be modified to address this effect. Studies such as this can help us to appreciate the unintended consequences of admissions processes and can identify ways to make the selection process more consistent, transparent and fair.

## Background

Admission to medical school is a serious matter for all parties involved. The task of selecting applicants who wish to become doctors is "a complex problem encumbered by inherent uncertainty" [1], and "daunting... selecting a small number of applicants who are most likely to succeed in medical school from a large pool of seemingly suitable applicants"[2]. Traditionally, selection has been based largely on academic performance, but recent work has focused on making the process more effective and representative of the needs of society [3,4] and has encouraged admissions committees to consider non cognitive attributes as well as academic performance [5].

Many medical schools utilize essay questions in student selection, and the essay question has long been included as a component of the Medical College Admission Test (MCAT). Literature on the use of the essay question in selection provides little consensus on its role; some suggest there is little evidence to support the continued use of essays [6], while others point out that essays complement selection based upon academic performance [7]. There is evidence that performance on an essay question reflects post-entry performance in applicants entering health-related professions [8], and that admissions essays scored for writing skills, clarity, organization and cohesiveness can predict academic success, and identify "problem students"[9]. A qualitative analysis of nursing school admission essays demonstrated that essays could differentiate between students who would complete their studies from those who would not, and that the essay question was a better predictor than measures of prior academic performance [10]. It has also been suggested that writing skills should be included in admissions processes because they are an essential part of healthcare education and practice [11,12].

The essay has been part of the selection process at our school for many years, being submitted along with transcripts, MCAT scores, and letters of reference to determine whether an applicant would be invited for interview. With the adoption of the multiple mini interview at our school [13,14], we also added two essays written in an invigilated setting. This study builds upon previous work in which we analyzed how applicants answered essay questions used in selection at our medical school [15], and was intended to provide a further exploration of observed patterns of applicant response. In that study, we concluded that applicants approached the essay questions as a way to tell their own subjective story; this approach was different from what was intended and expected by the school. The intent of this further study was to gain a better understanding of how applicants approached the essay questions utilized and to situate the findings in the context of the selection process as a whole.

## Methods

This study was based on the analysis of three essay questions utilized in the selection process at our school in 2007. The school is a three-year medical school which received a total of 1,476 applications in the year under study. From this group, 417 students were invited for interview, and 125 students were finally offered admission. Questions were developed by the Admissions Committee and were intended to test for traits of altruism, understanding of professionalism, and reflection/self-assessment (Table 1). The essay on volunteerism was submitted online before interview, with the question being posted three months before the application deadline, while the other two essays, on professionalism and reflection, were completed by applicants at the conclusion of the multiple mini-interview process. Each essay was approximately one page in length and was completed in 30 minutes. The online essay was scored by four reviewers, while the essays done at the time of interview were marked by a single assessor and made up a total of 10% of the score used for final ranking. No other writing by the applicant (eg. a personal statement) was included in the selection process.

**Table 1:** Essay Questions & Questions used in Interviews with Applicants and Assessors

Online essay on Volunteerism (intended to test for altruism)	Setting: Question posted online for 3 months before deadline, submitted with application.
	Question: *Why is volunteerism important, and how has your volunteer activity influenced you and developed your ability to become a medical doctor? The final paragraph should include some explanation of why you have decided on a career in medicine*.
Essay on Professionalism (intended to test for understanding of elements of professionalism)	Setting: Written at a computer after interview, 30 minutes allowed.
	Question: Applicants were presented with a short definition of professionalism and three statements describing specific professional behaviours from authorities such as the medical school or a medical College, and then asked: *What does this information tell you as an applicant to medical school?*

Essay on Reflection (intended to test for reflection and self-assessment)	Setting: Written at a computer after interview, 30 minutes allowed.
	Question: *You just completed an interview process where you had a chance to deal with the following stations. What did you learn about yourself during these 3 scenarios that might be important if admitted to our medical school? *(followed by information from three stations)

Questions used in interviews with applicants	• "how did you decide how to answer the essay questions?"
	• "were you able to answer the essay questions freely and honestly?"
	**"what effect did being in a competitive process have on your answers?"**

Questions used in interviews with assessors	• "how did you approach the job of marking?"
	**"did you think all of the material you read in the essays was truthful?"**

Essays for analysis were selected at random from the applicants invited for interview, and were anonymized before analysis commenced. The analysis was undertaken independently of the admission decisions process. All applicants gave permission for use of their written submissions for research purposes.

As this area of the literature is relatively under-examined, we chose to conduct an exploratory, qualitative approach to analyze the essays in order to derive a conceptual framework which would describe the patterns of response observed, and to express the relationships between the ideas and concepts encountered [16-18]. As our previous work has focused on the content of applicant responses ("what was said") [15], this study was intended to examine the patterns of applicant response in more detail ("how it was said") with the intent of determining the factors which influence applicant response ("why it was said").

In keeping with the qualitative methodology employed, it is important to note the perspective and intent of the study team involved in this work. The study was not designed to consider the value of the questions themselves, nor how they were created, or to develop "better" or "more objective" questions; this was the reason we did not adopt an approach based on linguistics or hermeneutics. Neither did we plan to apply a psychometric approach to determine the reliability or validity of the questions used in selection. Instead, the conceptual basis of this study was to observe an existing complex process *in situ*, with the aim of reaching a better understanding of how applicants approach selection for medical school, and then moving beyond the immediate context to situate these findings in the larger context of the admissions process as a whole.

Analysis was first conducted on 60 examples of each of the three essays selected at random, focusing on the patterns of writing observed in each essay. Essays were analyzed by a two readers (JW and JL) using iterative reading of texts, and selection of key words and phrases to develop a coding scheme in which the ideas expressed were categorized. Reflective journaling, charting and memo-writing were also used to record observations on the underlying themes in the data as analysis proceeded. Coding was reviewed, coding structures amended and debated, and consensus achieved at data analysis meetings held throughout the study period, and a theoretical explanation of the observed patterns of applicant response was generated. At the conclusion of analysis, 10 more examples of each essay were analyzed to ensure that data saturation (no further emergent themes) was achieved. The findings of this study are thus based on analysis of a total of 210 essays (60 + 10 for each of 3 essays).

After completion of analysis, we held interviews with a number of successful applicants and assessors who were involved in the admissions process in 2007. Convenience sampling was used; all applicants invited for interview had applied to the University of Calgary, and were offered admission either to the University of Calgary or the neighbouring University of Alberta. No unsuccessful applicants were contacted. Applicants were invited to participate by email; sessions were held with 20 applicants in two groups (60 minutes each) and with 4 assessors one-on-one (30 minutes each), and were tape-recorded and transcribed. We tested the generated theory and discussed the findings of analysis with these participants to gain another perspective on the role of the essay in the admissions process (participant checking/data triangulation). Questions used to stimulate discussion were generated from the results of analysis and are shown in Table 1. Ethical approval for the study was obtained from the University of Calgary Conjoint Health Research Ethics Board.

## Results

Analysis of the essays revealed that in many instances, material given in an answer did not seem to relate directly to the question provided, but appeared to have been included for another, unstated purpose. Answers included information which did not bear directly on the question asked, but seemed instead to be intended to impress the reader. For instance, in the question on volunteerism: "my GPA increased dramatically in the subsequent two years, ending with a 3.78/4 for my final year". Such examples were coded as "impressive details" and "impressive stories". Applicants also used their answers to express a sense of entitlement to admission of medical school (coded as "expressing expectations of reward") and to state that they were sure they were good enough to be admitted ("statements of confidence and destiny"). There were also many examples of applicants answering a question by restating and agreeing with the assumptions implicit in the question, as distinct from considering all aspects of the question asked (coded as "agreeing with the assumptions of the question"). Codes and representative quotations are presented in Table 2.

**Table 2:** Codes and Representative Quotations

"Impressive Details"	- I tutor my peers in self-fashioned strategies that have earned me a 4.42 (of 4.5) GPA and academic awards.
	- my GPA increased dramatically in the subsequent two years, ending with a 3.78/4 for my final year.
	- As the Vice President of the Student Government I have shouldered a lot of responsibility and have enjoyed being in that role.
**"Telling impressive stories"**	- The hardships I had to endure as a teenager have led me to pursue a career in medicine.
	- I never thought that I might have to go through the same things as the injury survivors until the day I was diagnosed with [an illness].
	- In the summer... I participated in a medical mission to [a country overseas]... I had worked in several clinics and hospitals prior to this particular trip, however, it was here that I believe the concepts of professionalism, honesty, and integrity truly shone...

**"Expressing expectations of reward"**	- I have served. - I have volunteered.--I have given of my time.
	- I have worked hard.--I have contributed.

**"Statements of confidence and destiny"**	- I have the confidence to say that I can be a professional physician.
	- These are professional codes which I will have no trouble abiding by as I exhibit professionalism in my daily activities.
	- I already possess many of the characteristics, skills, attitudes and behaviours which are necessary in the medical profession.
	- this information shows me that I will be chosen based on the required requirements of the profession of medicine.

**"Agreeing with the assumptions of the question"**	- I have no doubt that I would adhere to the confidentiality, integrity, and honesty that would be expected of me as a physician.
	- integrity, ethics and confidentiality are extremely important to me.

Further analysis focused on describing and explaining these patterns of response in more detail, with the purpose of understanding the factors which applicants considered when determining how to answer these essay questions. This process led to the development of a theoretical explanation named "What do they want me to say?" based upon the patterns of response observed in the data.

### Elaboration of theory

This theory proposes that considerations of "who will be reading my answer, and what do they expect me to say?" are at least as important to applicants as the content of the question itself. These effects are predicted to be especially relevant for a question with an "obviously correct" answer, in which applicants can easily divine and provide the "desired" response. These findings bring to light tension between "a genuine response" and "the expected response" that we believe applicants experience when choosing how to address an essay question in the admissions process. This point was probably best articulated in the following extract in which an applicant reflected on the difficulty of choosing between a "hypothetical and ideal protocol" (expected answer) and an "informal, genuine attitude" (a genuine answer): "Being graded on an interaction can be both a challenging experience and a rewarding one. It can be challenging in the sense that there is slight ambiguity as to how much one should try to follow some hypothetical and ideal protocol and how much one should maintain an informal and genuine attitude at the risk of compromising the latter formal aspect of a formal interview"

We propose two main factors which interact to determine how applicants decide "what do they want me to say?" when faced with a question in the selection process: 1: perceived expectations of the selection process and 2: ways of conforming to meet those expectations. We propose that expectations of the selection process are formed by obtaining information from other applicants, medical students and schools, and from the experience of prior application. We suggest that applicants consider a perceived 'acceptable' range of response, and ensure that their own response falls within this range. We detected much evidence that applicants include material expressly intended to enhance their chances of a successful admission to the school. This is considered to be the reason why applicants included statements of confidence and destiny ("I am ready"), and why they included impressive details ("I have a GPA of 4.0"). We also observed that most applicants did not include material likely to be perceived as unacceptable in the context of the selection process--no applicants gave answers disagreeing with the assumptions of the questions asked, suggesting some degree of self-censoring. As part of the theory generated, we also proposed that applicants would be expected to consider the behaviour of other applicants when deciding how to respond to a particular question.

### Confirmation of theoretical explanation by participant checking/data triangulation

**Figure 1:**
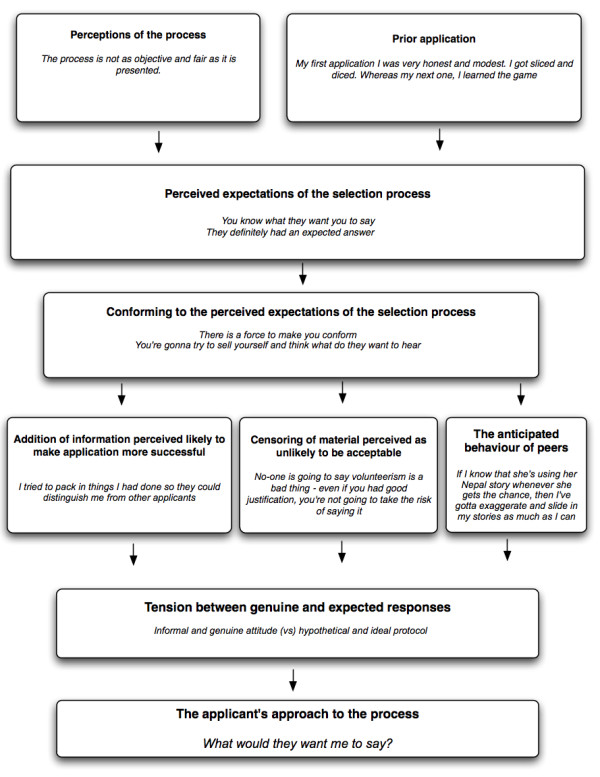
**The theoretical explanation "What Do They Want Me to Say?"**.

The elements of "What Do They Want Me to Say?" were supported by interviews with applicants and assessors (Figure 1 and Table 3). Applicants expressed a clear, shared understanding of the 'proper' answers expected by the school, and described modulating their responses to conform with their perceived expectations of the selection process. One applicant described her understanding of the school's expectations very succinctly: "I did that constantly through the entire interview process, thinking: "What would they want me to say?" One applicant described applying for medical school and "getting sliced and diced" because he did not understand "the unwritten rules" of the process in the same way as the other applicants. There was also discussion about the need to "sell yourself" in the application process, using terms such as "standing out" and "making your answer different". Applicants admitted behaviors such as "exaggerating" and "polishing", and gave accounts of having prepared answers, and repeating stories perceived to be effective. At interview, applicants described these behaviors as acceptable practice. Assessors also expressed concerns about exaggeration, and described essays which appeared to have been written with the clear intent of impressing the assessor.

**Table 3:** Quotations from Applicants & Assessors

Quotations from Applicants	Perceptions of the process
	The process is not as objective and fair as it is presented.
	The way to beat a random process is to increase your numbers.
	Effect of Prior Application
	My first application I was very honest, and modest. I got sliced and diced. Whereas my next one, I learned the game, I definitely exaggerated, it would hold up in a court of law, but it was completely exaggerated.
	Are you just getting better at going through an admissions process or are you becoming a better candidate?
	Perceived Expectations of the Process
	In the professionalism essay, they definitely had an expected answer--because, in hindsight, I don't think I wrote what they wanted.
	Conforming to the Perceived Expectations of the Process
	I do agree that there is a force to make you conform--I'm not an A Type personality, but I became one in premed and that's what stressed me out.
	You think about what they would want to hear and try to use it to your advantage... you're gonna try to sell yourself and think what do they want to hear.
	Volunteerism was the topic, but my approach was: this is a chance to sell myself. Bottom line: I didn't really think of it as an essay--it was more like "How can I adapt why I would be a good candidate into this essay and mould it into the topic that they gave me?" You sell yourself and want them to want you.
	Addition of information perceived likely to make application more successful
	I tried to pack in things I had done so they could distinguish me from other applicants.
	I definitely exaggerated.
	You only have that chance to say one thing, so you want to say one thing that will make an impact.
	Censoring of material perceived a unlikely to be acceptable
	- You know what they want from that question--volunteerism is a good thing. No-one is going to say volunteerism is a bad thing.
	- That's not what they want to hear--even if you had good justification, you're not going to take the risk of saying it.
	The anticipated behaviour of peers
	- A lot of people don't take it as seriously as others. I know people who haven't prepared, who don't realise how much effort other people put into it. If they did realise it, they would obviously prepare more.
	- If I know that she's using her Nepal story whenever she gets the chance, then I've gotta exaggerate and slide in my stories as much as I can.
	**Quotations from Applicants contd**.
	Tension between genuine and expected responses
	- There's like the truth, and then people tend to embellish--you'll bring certain things to the forefront that, maybe you didn't spend as much time doing, but that's like your only volunteer thing, so you're going to really like bring up, so you're not necessarily lying.
	The applicant's approach
	- I did that constantly through the entire interview process, thinking: "What would they want me to say?"
**Quotations from Assessors**	**Conforming to expectations**
	- They know that you have the power, they know their job is to impress you, whatever it takes. All that matters is that I get in here. I don't care if I'm true to anything, I'll worry about that stuff later.
	- There's so much at stake for people to be dishonest or use whatever means... and I know the medical students get together to coach next year's group on what to say--you know like the Olympics and drug doping--like we're always trying to outsmart them or whatever and I don't know even the right way at all.
	**Concerns about exaggeration & truthfulness**
	- The impression I got was applicants were exaggerating--you read their accounts and got quite cynical... it goes against the grain of what a young doctor should be.
	-I think that honesty doesn't even come into it, because the stakes are so high. "I sold my soul last night, but you know what, I'm gonna help people in surgery, so I'll make it up there, I'll buy it back". I think our system forces people to do that.

## Discussion

This work demonstrates that that the process of selection has a strong influence on how applicants respond to questions used in medical school admissions. Applicants were observed to be conforming to the expectations of the selection process in ways intended to maximize their chances of acceptance. Applicants admitted to giving answers perceived to be the most acceptable to the school, and to having a shared understanding about the "unwritten rules" of acceptable conduct in the process. These findings suggest the existence of a "hidden curriculum" at work in the admissions process, by which applicants understand how they must behave to gain entry. This parallels a similar phenomenon previously noted in premedical education and beyond [19-23] which has been described as "a set of influences that function at the level of organizational structure and culture", "commonly held understandings... and taken-for-granted aspects of what goes on", "implicit messages... about what the institution considers important" and "implicit rules (used) to survive the institution" [21,24]. In this case, we propose a set of influences arising from the selection process through which applicants gain an understanding of what the school desires and how applicants must behave in order to gain admission. Some of the "unwritten rules" of this hidden curriculum may be similar to those expressed by applicants in this study: "figure out what they want to hear", "sell yourself--make them want you", "exaggerate but don't get caught", "do whatever you have to do to get in".

Gaining admission to medical school is highly competitive: pre-medical education has been described as "a series of obstacles to be overcome on the way to the elusive goal of medical school admission" [19], with applicants possessing "a fiercely contentious desire to prevail over others, and at all costs get into this or that or any medical school" [25]. As pointed out in a recent review, this competitiveness has an effect on the selection process: "Trust no-one. Do not trust the applicants. When stakes are high, the likelihood of cheating increases; the stakes for medical school admission are very high" [26]. Others have observed medical school applicants "buffing up the application form" [27], and similar phenomena have been noted in the selection process for residency [28].

A number of theories of human psychology are relevant to our findings. Social learning theory suggests that applicant response will be determined by consideration of expectations, consequences, rewards and punishments occurring within a complex social system [29]. Theories of impression management and self-presentation also relate to our work, as they describe goal-directed conscious or unconscious processes in which people attempt to influence the perceptions of others by regulating and controlling information in social interaction [30]. Work on social desirability bias also bears on our findings, as it describes the tendency of respondents to reply in a socially-acceptable manner that will be viewed favorably by others, instead of responding with their own opinions, a phenomenon known as opinion conformity [31]. Applicants may infer from a question a socially-desirable "expected response" with which they are required to conform in order to obtain reward (admission to medical school) [32]. Conforming to the expectations of the application process may well be the first step in a longer, more sustained process of conforming which occurs throughout medical school and beyond. As Coles states: "we let (students) know by our admissions practices what kind of person we desire" [25]. It is interesting to speculate that problems relating to unprofessional behaviour may originate in messages sent to applicants at their entry into medical education [33,34].

It can be argued that behaviours such as "exaggeration" and "selling" are simply an accepted part of any competitive selection process which operates "in the real world", although such behaviors have not been acknowledged in the literature on medical school admissions to date. It may be that we expect applicants to be "responsible for knowing when (and how) to break the rules" [35]. Such an approach creates inherent unfairness and bias against those who are operating under different assumptions, and creates a dissonance between what schools intend and what is experienced by applicants [36]. It is important to remember that applicants are only responding to the pressures of selection created by the processes that schools have chosen to employ [32,36].

Selection might be improved by taking steps to make the "hidden curriculum of admissions" more explicit [23]. Addressing pre-medical culture and preparation is important [19], and we suggest the selection process should include discussion about applicants' perceptions of the pressures of the process, and about the conflict between needing to "sell yourself" while trying to remain genuine [23]. Steps such as these may assist in demystifying what is a high-pressure process, and may reduce anxiety and dispel incorrect beliefs about the process obtained from the "admissions rumor mill" [3,20,28,32,34,36]. The nature of the questions used in selection is also important: other studies have suggested that self-declared values should not be used in admissions decisions [37], a finding very much in keeping with our core variable "What do they want me to say?" Answers to questions about the self should be treated with much caution in the selection process; as Siu and Reiter advise: "Avoid self-reporting... to expect the bad apples to weed themselves out on the basis of self-reporting goes beyond Pollyanna into self-delusion. All observations about the candidate should be made from a more remote position than the applicants themselves" [26].

This study was limited as it considered the selection process at only one school in one year, it examined essays written both online and in person, and involved only a subset of applicants and assessors in interview. We hope that our findings will spur other investigators to consider this under-represented part of the literature, and to determine if these effects can be observed elsewhere.

Our observations on the effects of the selection process are expected to be provocative, as they reveal a side of the admissions process which appears well-understood by applicants but which has not been reported in the admissions literature to date. We anticipate that this study will make uncomfortable reading for those engaged in the admissions process, as it considers the acceptability of the behaviours which are required to gain entry to medical school. As Hafferty has written: "until we come to accept that medical training is, at root, a process of moral enculturation and... medical schools function as moral communities, the reform that is needed--the reform that the public deserves--will remain both elusive and enigmatic" [21].

## Conclusions

This work suggests the existence of a "hidden curriculum of admissions", and demonstrates that the process of selection for medical school has a strong influence on applicant response. Our findings also highlight the social context of the admissions process, with applicant response being affected by factors such as expectations, consequences, rewards and punishments. We suggest two ways in which the selection process might be modified to understand and address this effect: discussing the hidden curriculum of the admissions process more openly, and avoiding questions which ask directly about the self. Studies such as this can help us to appreciate the unintended consequences of admissions processes and can identify ways to make the selection process more consistent, transparent and fair.

## Competing interests

The authors declare that they have no competing interests.

## Authors' contributions

JW and JL conceived the original idea for the study and were responsible for the study design. JW and JL carried out the primary data analysis. JL and KB reviewed the results of primary analysis and assisted with construction of the discussion. All authors contributed to, read and approved the final manuscript.

## Authors' information

Dr Jonathan White MD PhD is an Associate Professor and Tom Williams Endowed Chair in Surgical Education in the Department of Surgery at the University of Alberta. Dr Keith Brownell MD is a Professor in the Departments of Clinical Neurosciences and Medicine at the University of Calgary. Dr Jean-Francois Lemay MD is an Associate Professor in the Department of Pediatrics at the University of Calgary. Dr Jocelyn M Lockyer PhD is a Professor in the Department of Community Health Sciences and is Associate Dean for Continuing Medical Education and Professional Development, University Of Calgary.

## Ethical approval

University of Calgary Conjoint Health Ethics Review Board.

## Published in abstract form

White JS, Lockyer J. "What Do They Want Me to Say?" A Qualitative Analysis of the Use of the Essay Question in Medical School Admissions (abstract), *Medical Education *2009, 43(Supp1): 27

## Pre-publication history

The pre-publication history for this paper can be accessed here:

http://www.biomedcentral.com/1472-6920/12/17/prepub
